# Testicular and ovarian Juvenile granulosa cell tumors in children and adolescents: Analysis of 113 patients registered to the German Registry for Rare Pediatric Tumors (STEP)

**DOI:** 10.1002/cncr.35861

**Published:** 2025-04-24

**Authors:** Dominik T. Schneider, Andrea Witowski, Michael Abele, Martin Benesch, Benedikt Bernbeck, Tabea Blessing, Bastian Brummel, Gabriele Calaminus, Ulrich Göbel, Norbert Graf, Christian Vokuhl, Kris Ann P. Schultz, Ines B. Brecht

**Affiliations:** ^1^ Clinic of Pediatrics Klinikum Dortmund University Witten/Herdecke Dortmund Germany; ^2^ Department of Pediatrics and Pediatric Hematology and Oncology University Hospital Tubingen Germany; ^3^ Division of Pediatric Hematology/Oncology Department of Pediatrics and Adolescent Medicine Medical University of Graz Graz Austria; ^4^ Clinic of Pediatric Hematology and Oncology University Bonn Bonn Germany; ^5^ Heinrich‐Heine‐University Dusseldorf Dusseldorf Germany; ^6^ Clinic of Pediatric Hematology and Oncology Saarland University Homburg/Saar Germany; ^7^ Department of Pediatric Pathology University Bonn Bonn Germany; ^8^ Cancer and Blood Disorders Children's Minnesota Minneapolis Minnesota USA

**Keywords:** juvenile granulosa cell tumors, pediatric oncology, sex cord stromal tumors, very rare tumors

## Abstract

**Background:**

In juvenile granulosa cell tumors (juvGCTs), impaired survival was reported after preoperative tumor rupture, peritoneal metastases, or high mitotic rate (≥20 mitoses per 10 high‐power fields). Therefore, a risk stratification was developed to select patients for chemotherapy.

**Methods:**

Between 2001 and 2019, 89 female patients and 24 male patients were prospectively enrolled. Histopathologic classification was according to the World Health Organization classification, and staging was according to Children's Oncology Group and International Federation of Gynecology and Obstetrics classification.

**Results:**

Testicular juvGCTs were detected as scrotal swelling during infancy. No recurrences were reported after orchiectomy. Patients with ovarian juvGCTs presented at a median age of 9.8 years with abdominal discomfort, isosexual precocity, or amenorrhea. After tumor resection, two of 52 patients with stage IA disease, one of 14 with stage IC1 disease (intraoperative rupture), 13 of 18 with stage IC2 or IC3 disease (preoperative rupture), and all five patients with stage II/III disease received chemotherapy. Four recurrences with two deaths were reported. Three recurrent tumors were initially stage IA with a high mitotic rate, and one was a stage II tumor. No recurrences were observed among patients who had stage IC2/IC3 disease, who had unfavorable prognoses in historical cohorts. The 5‐year event‐free survival was 0.95 ± 0.03 (85 of 89 patients), and overall survival was 0.97 ± 0.02 (87 of 89 patients).

**Conclusions:**

Testicular and ovarian juvGCTs are clinically distinct entities. Although testicular juvGCTs exclusively present during infancy and have an excellent prognosis, ovarian juvGCTs may arise at any age and constitute potentially aggressive tumors. Centralized reference diagnostics and the establishment of counseling structures for the treatment of patients with ovarian juvGCTs improved prognosis compared with historical groups. The mitotic rate and incomplete surgery were identified as important risk factors in addition to tumor stage and should be considered in the risk‐stratification of therapy.

## Abstract 2

Although testicular juvenile granulosa cell tumors exclusively present during infancy and have an excellent prognosis, ovarian tumors may arise at any age and constitute potentially aggressive tumors. In patients with advanced‐stage tumors, adjuvant cisplatin‐based chemotherapy may reduce the risk of progression.

## INTRODUCTION

Juvenile granulosa cell tumors (juvGCTs) belong to the heterogeneous group of rare sex cord stromal tumors.^1^, ^2^, ^3^ Altogether they account for approximately 5% of all gonadal cancers in childhood and adolescents. JuvGCTs are an important differential diagnosis to the more frequent gonadal germ cell tumors and epithelial ovarian cancers.^4^ According to their name, granulosa cell tumors derive from the granulosa cells of the ovary, the so‐called follicular epithelium. Nevertheless, juvGCTs may arise on both testis and ovary. Physiologically, granulosa cells surround the oocyte and may produce estradiol from puberty onward. According to their physiologic correlate, juvGCTs also may be associated with estradiol secretion. Going along with the production of estrogen, juvGCTs may also secrete inhibin, which can be used as a serologic and immunohistochemical marker.^5^

JuvGCTs are distinguished from adult granulosa cell tumors by their specific histologic pattern and also show characteristic clinical features.^2^ Thus, in contrast to adult granulosa cell tumors, they may arise in both testes and ovaries, they are not prone to late relapses, they may show high mitotic activity, and they do not or only rarely display *FOXL2* mutations.^6^

Like other very rare tumors, juvGCTs have been orphan diseases for a long time. Because of their rarity, no randomized clinical trials have been established for juvGCTs; indeed, such an endeavor may be futile, even in international networks. This is explained by the low incidence of juvGCTs in general and the comparably low percentage of patients with an unfavorable risk profile who require adjuvant therapy beyond tumor resection. Most clinical series published to date have been generated from national registries for very rare tumors in childhood, or they have been documented along with clinical trials for pediatric germ cell tumors.^7^, ^8^, ^9^ Consequently, uncertainty remains regarding the optimal therapeutic approach to these patients, in whom a significant risk of tumor progression is expected.

Several cohort studies have shown that the prognosis of testicular juvGCTs is favorable, and to date, no fatal courses have been reported.^10^, ^11^, ^12^, ^13^ In contrast, ovarian juvGCTs may be diagnosed with a considerable size, and may even present with spontaneous tumor rupture and metastatic spread and lead to an ultimately fatal course. In 2003, the analysis of a prospective case collection of 54 ovarian sex cord stromal tumors, including 45 juvGCTs reported to the German MAKEI (Maligne Keimzelltumoren) study group on childhood germ cell tumors, was published.^14^ Briefly, patients with spontaneous tumor rupture or metastases to the pelvis or abdomen had a risk of tumor progression ≥50%r.^15^ Based on this analysis, a risk stratification has been developed that considers tumor stage, including the time of potential tumor rupture (preoperative vs. intraoperative rupture), as well as histologic parameters, such as mitotic activity. This risk stratification has been established in Germany since 2001, and it has been the basis of any consultation work of the German Registry for Rare Pediatric Tumors (STEP) for patients with sex cord stromal tumors.^16^

Here, we report on the prospective series of 113 patients with juvGCTs, including 89 ovarian and 24 testicular juvGCTs, who were treated according to this risk stratification. We present the clinical data from this cohort, summarize therapeutic data, and compare the therapeutic strategy and outcomes with those of the historic cohort. The objective of the analysis presented here is to evaluate the risk‐stratification strategy in a large cohort of prospectively enrolled patients.

## MATERIALS AND METHODS

### Patients

Since implementation of the MAKEI 96 trial, the coordination center has provided clinical advice for patients with rare gonadal tumors including sex cord stromal tumors and has prospectively collected clinical data of these patients, who have been categorized as observational follow‐up patients. This work was coordinated by three of the authors (D.T.S., U.G., and G.C.). After establishment of the STEP working group in 2006, the clinical consultation work has been coordinated within the STEP network (D.T.S. and I.B.), and clinical data have been prospectively included in the STEP registry (ClinicalTrials.gov identifier NCT05773651). All patients were treated in pediatric oncological centers in Germany, Austria, and Switzerland.

In total, 119 patients with juvGCTs were prospectively registered between 2001 and 2019. After the exclusion of patients older than 18 years, 113 tumors were included in this analysis (24 testicular and 89 ovarian juvGCTs). The median follow‐up was 95 months (range, 1–155 months) for testicular juvGCTs and 53 months (range, 1–179 months) for ovarian juvGCTs.

### Data collection

Patient data were prospectively collected and documented on standardized data documentation forms, which included epidemiologic, anamnestic, clinical, diagnostic, and pathologic data. Reference pathology was obtained from the German Childhood Tumor Registry at the Universities of Kiel and Bonn, respectively (C.V.). Clinical, surgical, and pathologic data were reviewed at the coordination center. A central consultation process after data review was provided to the treating physicians, including a written recommendation regarding therapy and follow‐up.

### Staging and histopathologic classification

Patients were diagnosed according to the guidelines for testicular and ovarian germ cell tumors, respectively, including measurement of the tumor markers α‐fetoprotein (AFP) and β‐human chorionic gonadotropin (β‐HCG). Imaging included ultrasound and abdominal/pelvic MRI. A chest CT was not recommended. Testicular tumors were staged according to the Children's Oncology Group (COG) staging system, and ovarian tumors were classified according to the (updated) International Federation of Gynecology and Obstetrics/World Health Organization (FIGO/WHO) system for ovarian cancer.^17^ Intraoperative staging procedures included the collection of peritoneal fluid or washing for cytologic assessment before manipulation of the tumor. Histopathologic classification of the tumor was done by the national reference pathologists (C.V.) according to the WHO classification and previous reports by the study group led by Young and Scully.^18^

### Treatment and follow‐up data

All patients underwent surgical treatment according to the current recommendations for malignant germ cell tumors. Thus, in most patients, inguinal orchiectomy, ovariectomy, or adnectomy was performed. There were no standardized treatment protocols for adjuvant treatment, but the choice of treatment was the responsibility of the treating physician. During the consultation between treating physician and coordination center, the risk‐stratification strategy implemented after the previous MAKEI analysis^14^ as well as data from other international study groups^10^, ^19^, ^20^, ^21^ were provided, and a written recommendation with regard to therapy stratification as well as staging and follow‐up investigations was issued. As a general recommendation, all stage I testicular tumors were followed expectantly. Most patients with FIGO stage IA tumors and those with intraoperative spillage (FIGO stage IC1) also were followed expectantly. In case of preoperative rupture (stage IC2), microscopic metastases (stage IC3), or macroscopic metastases (II, III), adjuvant chemotherapy analogous to the MAKEI 96 protocol was recommended. This included cisplatin (100 mg/m^2^ per cycle), etoposide (300 mg/m^2^ per cycle), and ifosfamide (7500 mg/m^2^ per cycle; PEI), with concomitant infusion therapy as described elsewhere.^22^ Up to four cycles of PEI were recommended. If the tumor was resected incompletely (R2) or if there were peritoneal or lymph node metastases, a second‐look surgery with resection of all visible tumor remnants and metastases was recommended upon consultation.

Treatment data included the type and completeness of tumor resection and the type and amount of chemotherapy or radiotherapy. Follow‐up data included the time of last‐follow‐up, remission and survival status, and information regarding secondary disorders. In case of relapse, all available information regarding salvage therapy was documented.

### Statistical analysis and ethical issues

After pseudonymization, data were collected within an ACCESS database using a protected hospital server (with access limited to authors A.W., T.B., B.B. and D.T.S). Because of the small cohort of patients, statistical data analysis was mainly descriptive using the SPSS version 26 statistical software package (IBM). Categorical data were compared using χ^2^ tests, and numerical data were compared using the Mann–Whitney *U* test. Survival analysis was done according to the Kaplan–Meier method, and differences between subgroups were evaluated using the log‐rank test. For survival analysis, metachronous tumors were counted as events but not relapses.

All patients provided informed consent to central data collection in the STEP and MAKEI registries. The local ethics committees at the universities of Dusseldorf, Erlangen, and Tubingen approved the clinical registries and studies.

## RESULTS

### Testicular juvenile granulosa cell tumors

All 24 patients included were neonates or infants, with a median age at diagnosis of 16 days, ranging from the first day of life until 12 months. In 17 patients, the tumor was diagnosed in the first month of life; and, in seven patients, the tumor was detected at an age from 6 to 12 months. There were 13 left‐sided and 10 right‐sided tumors as well as one patient with bilateral tumors. Tumors were detected as painless scrotal swelling with no clinical signs of hormone secretion. The greatest tumor dimension ranged from 2 to 6 cm (median, 2 cm), with no correlation between age at diagnosis and greatest tumor dimension. Tumor markers (AFP and β‐HCG) were within age‐related, normal reference levels. All but one patient with unilateral tumors underwent complete orchiectomy, and one patient underwent organ‐sparing partial orchiectomy. The patient with bilateral, diffuse juvGCTs was treated with luteinizing hormone‐releasing hormone (LH‐RH) blockage using buserelin, and he was histologically confirmed tumor free after 14 months. All tumors were stage I; no lymph node or organ metastases were detected. After a median follow‐up of 95 months (range, 1–155 months), no relapse was observed.

### Ovarian juvenile granulosa cell tumors

#### Clinical presentation

The median age of the 89 patients was 9.8 years (range, birth to 18 years). Seven patients were diagnosed during the first year of life. Thirty‐nine patients (44%) were younger than 8 years at diagnosis and thus prepubertal, whereas 50 patients (56%) were older than 8 years. Therefore, patients with ovarian juvGCTs were significantly older than their counterparts with testicular juvGCTs (*p* < .01). Clinical signs at presentation in 85 patients who had complete clinical data are summarized in Table 1. The most frequent complaints included abdominal discomfort or pain (56%) and, in some patients, even acute abdomen because of ovarian torsion, as well as abdominal swelling (42%). Notably, isosexual precocity was the leading symptom in 71% of prepubertal patients, whereas symptoms related to abnormal hormone secretion (e.g., secondary amenorrhea) were less frequently observed in pubertal or adolescent girls.

**TABLE 1:** Symptoms in ovarian juvenile granulosa cell tumors correlated with age (85 patients with complete data).

Symptoms	No. (%)		
	Younger than 8 years, *n* = 38	Aged 8 years and older, *n* = 47	Total, *n* = 85
Abdominal pain	13 (34)	35 (74)	48 (56)
Ovarian torsion	4 (11)	1 (2)	5 (6)
Abdominal swelling	13 (34)	23 (49)	36 (42)
Pubertal precocity	27 (71)	—	27 (32)
Abnormal vaginal bleeding	11 (29)	13 (28)	24 (28)
Secondary amenorrhea	—	7 (15)	7 (8)
Hirsutism	2 (5)	1 (2)	3 (4)

Forty‐seven patients presented with unilateral left‐sided tumors, 41 presented with right‐sided tumors, and one patient presented with bilateral tumors. The median greatest tumor dimension was 11 cm and ranged from 2 to 30 cm. Correlated with age, tumors were smaller (median greatest dimension, 7.8 cm) in prepubertal children compared with pubertal adolescents (12.6 cm; Mann–Whitney *U* test; *p* < .0001). We observed a significant correlation between greater tumor size and the frequency of abdominal pain, but not with endocrinologic signs. Finally, tumor size did not correlate with tumor stage or outcome.

Among our patients, we did not detect secretion of AFP or β‐HCG. Inhibin B was measured preoperatively in only 11 patients and levels varied between 10 and 2284 ng/L. In five patients, significantly elevated inhibin B levels >1000 ng/L were measured, and three of these patients exhibited clinical isosexual pubertal precocity. Inhibin B levels normalized after tumor resection, and clinical signs of precocity regressed accordingly.

#### Tumor stage and histopathology

Eighty‐four patients presented at FIGO stage I disease, including 52 (58%) with completely resected stage IA tumors and 32 (36%) with intraoperative spillage, preoperative tumor rupture, or malignant ascites (Table 2). Accordingly, 14 patients were assigned to stage IC1, 14 were assigned to stage IC2, and four were assigned to stage IC3. Five patients (6%) had macroscopic peritoneal spread at diagnosis (four at stage II and one at III). Peritoneal metastases were most frequently detected in the pouch of Douglas, the pelvic wall, including infiltration of the sigma, and the uterus. The patients with stage III disease displayed metastases in the pelvis and the omentum. No distant or lymph node metastases were described at diagnosis.

**TABLE 2:** International Federation of Gynecology and Obstetrics stage of ovarian juvenile granulosa cell tumors correlated with age.

		No. (%)		
FIGO stage		Younger than 8 years	Aged 8 years and older	Total
IA		28 (72)	24 (48)	52 (58)
IC	IC1	7 (18)	7 (14)	14 (16)
	IC2/IC3	4 (10)	14 (28)	18 (20)
II/III		0 (0)	5 (10)	5 (6)
Total		39 (100)	50 (100)	89 (100)

Abbreviation: FIGO, International Federation of Gynecology and Obstetrics.

In 85 of 89 patients, a reference pathology evaluation was obtained. Thirty‐eight percent of tumors had a low mitotic rate (<10 mitoses per 10 high‐power fields [HPF]), 26% had a moderate mitotic rate (10–19 mitoses per 10 HPF), and 36% had a high mitotic rate (>19 mitoses per 10 HPF). The median mitotic rate was 14 per 10 HPF, with a range of 1–72 mitoses per 10 HPF. Proliferative activity was also accessed by Ki‐67 (23 patients) or MIB‐1 (39 patients) immunohistochemistry, which demonstrated a highly variable proportion of staining, ranging from 5% to 8%, and no clear statistical correlation with the mitotic count. There was no statistical correlation between mitotic activity, proliferation staining, and tumor stage.

#### Therapy

In all patients with ovarian juvGCTs, tumor resection was the first therapeutic step and was performed through laparotomy in 75 patients (86%) and laparoscopy in 12 patients (14%; two patients had incomplete data). Fifty‐six patients (64%) underwent unilateral adnectomy with removal of the ovary and Falliopian tube, 24 (27%) underwent tumor ovariectomy, and eight (9%) underwent organ‐sparing tumor resection. In two patients, suspicious retroperitoneal lymph nodes were removed during surgery. In another three patients, metastases at the pelvic wall were biopsied or completely removed; and in one patient, the omentum was removed.

In 52 patients (58%), the resection status was considered microscopically complete (R0); and in 36 patients (41%), the resection was categorized as microscopically incomplete (R1; one patient had incomplete data). We observed no significant correlation between surgical approach (open vs. endoscopic) and completeness of tumor resection.

Twenty‐one patients (24%) received adjuvant chemotherapy, whereas 67 (76%) were followed expectantly at 3‐month intervals. All but one patient received PEI chemotherapy, similar to the MAKEI 96 protocol, and one patient received bleomycin, etoposide, and cisplatin. In one patient, cisplatin was replaced with carboplatin because of ototoxicity. One patient underwent stem cell apheresis followed by dose‐intensified PEI and stem cell transplantation. Among patients with stage I disease, one patient received three cycles of chemotherapy, five patients received four cycles, and one patient received six cycles. Among the five patients with stage II/III disease, one received one cycle of PEI followed by three cycles of dose‐intensified PEI; three received four cycles of PEI; one received four cycles of cisplatin, etoposide, and cyclophosphamide (instead of ifosfamide); and the last patient received six cycles of PEI (Table 3).

**TABLE 3:** Adjuvant chemotherapy correlated with tumor stage in ovarian juvenile granulosa cell tumors.

		No. (%)			
FIGO stage		W&W	Adjuvant chemotherapy	Incomplete data	Total
IA		50 (96)	2 (4)		52
IC	IC1	13 (93)	1 (7)		14
	IC2/IC3	4 (22)	13 (73)	1 (6)	18
II/III		0 (0)	5 (100)		5
Total		67 (75)	21 (24)	1 (1)	89

Abbreviations: FIGO, International Federation of Gynecology and Obstetrics; W&W, watch‐and‐wait strategy.

#### Follow‐up, outcome, and prognostic markers

After a median follow‐up of 53 months (range, 1–179 months), four relapses and two lethal events were reported. Thus 5‐year event‐free survival according to the Kaplan–Meier method was 0.95 ± 0.03 (85 of 89 patients), and overall survival was 0.97 ± 0.02 (87 of 89 patients). Of note, three relapses occurred in patients who had tumors initially classified as stage IA, and only one patient with a recurrent tumor had previously been diagnosed at stage II (Table 4). Details regarding their initial presentation, first‐line and relapse treatments, and outcomes are summarized in Tables 5 and 6. The tumors in all four patients exhibited high proliferative activity, illustrated by a high mitotic count ranging from 20 to 67 mitoses per 10 HPF, or a high proportion of Ki‐67–stained cells. In one patient (patient 2), vascular invasion was noted. Three of four patients underwent organ‐sparing surgery (enucleation of the tumor or partial ovariectomy).

**TABLE 4:** Tumor stage, adjuvant chemotherapy, and outcome in ovarian juvenile granulosa cell tumors.

		No. of patients					
		W&W		Chemotherapy		Incomplete data	Total
FIGO stage		First CR	Relapse	First CR	Relapse		
IA		48	2	1	1		52
IC	IC1	13	0	1	0		14
	IC2/IC3	4	0	13	0	1	18
II/III		0	0	4	1		5
Sum		65	2	19	2	1	89

Abbreviations: CR, complete remission; FIGO, International Federation of Gynecology and Obstetrics; W&W, watch‐and‐wait strategy.

**TABLE 5:** Initial presentation of four patients with ovarian juvenile granulosa cell tumors and consecutive relapses.

Patient	Age, years	Clinical presentation^a^	FIGO stage	Greatest tumor dimension, cm	Surgical therapy	Resection status	Proliferation indices
1	13.41	1, 2	IA^b^	10	Laparoscopic partial ovariectomy	R0	>20 mitoses per 10 HPF; MIB‐1 index, 30%
2	4.57	2, 3, 4	IA^c^	11	Open partial ovariectomy	R0	67 mitoses per 10 HPF
3	14.05	1, 2	IA^d^	21	Open unilateral adnectomy	R0	20 mitoses per 10 HPF; MIB‐1 index, 20%
4	10.65	1, 2	IIB	20	Tumor enucleation	R1	High mitotic rate; Ki‐67 index, 50%

Abbreviations: FIGO, International Federation of Gynecology and Obstetrics; HPF, high‐power fields; R0, microscopically complete resection; R1, microscopically incomplete resection.

^a^ Column values: 1 = pain, 2 = abdominal swelling, 3 = premature puberty, 4 = vaginal bleeding.

^b^ Gross ascites without cytologic evidence of tumor cells.

^c^ Vascular invasion.

^d^ With areas of anaplasia.

**TABLE 6:** Initial and salvage therapy after relapse of ovarian juvenile granulosa cell tumors and outcome after relapse.

Patient	Adjuvant therapy	EFS, months	Salvage therapy	Status	Survival after relapse, months
1	W&W	6.4	Surgical resection, 4 × PEI	Second CR	32.2
2	4 × PEI	12.6	Surgical resection, 1 × BEP, irradiation (30.6 Gy), 2 × JEB, autologous stem cell transplantation^a^	Third CR	126.9
3	W&W	16.0	Surgical resection, 1 × PEI^b^	DOD	19.5
4	4 × PEI	5.7	4 × carboplatin, paclitaxel, and bevacizumab; regional deep hyperthermia^c^	DOD	2.5

Abbreviations: BEP, bleomycin, etoposide, and cisplatin; CR, complete remission; DOD, died of disease; EFS, event‐free survival; Gy, grays; JEB, carboplatin, etoposide, and bleomycin; PEI, cisplatin, etoposide, and ifosfamide; W&W, watch‐and‐wait strategy.

^a^ Second relapse after 6.9 months in paratracheal lymph node, treated with resection, bevacizumab, paclitaxol, thalidomide, and pegylated interferon.

^b^ Chemotherapy was discontinued upon patient’s request after 1 × PEI; start of PEI chemotherapy and regional deep hyperthermia at second tumor progression; palliative chemotherapy with thalidomide, vinblastine, topotecan, etoposide.

^c^ Progression during salvage chemotherapy and one cycle of regional deep hyperthermia; palliative therapy.

In the analysis of prognostic factors, it must be noted that all four patients who had recurrent tumors suffered from tumors that had high mitotic activity. Accordingly, the probability of EFS of patients who had a low or moderate mitotic rate (<20 mitoses per 10 HPF) was 1.0 (61 of 61 patients); whereas, in patients who had tumors with high mitotic activity (>19 mitoses per 10 HPF), the probability of EFS was 0.85 ± 0.07 (24 of 28 patients; log‐rank *p* = .032). In contrast, we did not observe a statistically significant correlation between tumor stage and outcome; however, patients who had higher stage disease received chemotherapy significantly more often than those with stage IA disease (*p* < .01).

## DISCUSSION

Similar to many very rare tumors, no prospective therapeutic or even risk‐stratified or randomized clinical trials are realistic for sex cord stromal tumors. However, even taking into account the rarity of events, valid treatment recommendations can be developed if patients are diagnosed according to high standards and if treatment recommendations are harmonized in networks so that recurring patterns can be identified. In the previous analyses of cohorts derived from the MAKEI study, tumor site, stage, and histologic parameters like such as the mitotic rate emerged as the most important prognostic factors for patients with ovarian juvGCTs, whereas patients with testicular juvGCTs generally fared well.^12^, ^14^ The analysis presented here builds on these previous works. It reflects the growing diagnostic and consultation network that has been established in Germany to optimize diagnostic assessment and therapy stratification.

All patients have been diagnosed according to best available standards, including central pathology review. If reported to the STEP registry in a timely manner, all patients have been reviewed by the study group at a consultation board, and therapeutic recommendations have been provided according to uniform standards, recommending adjuvant chemotherapy for patients with ovarian stage IC2 tumors and higher stages. In general, we have observed a good adherence to these recommendations (Table 3). Because of the high diagnostic standard, the uniform clinical assessment, and therapy according to harmonized standards, meaningful and valid conclusions can now be drawn from this updated cohort.

Of note, even for rare tumors, registration rates can be increased significantly by installing central reference institutions. Compared with the early registration period in the MAKEI study, we have observed an increase in the annual registration rate of low‐stage tumors. Consequently, this cohort constitutes the world‐wide largest prospective cohort of pediatric juvGCTs collected to date.

First, it becomes obvious that testicular juvGCTs have a clinical presentation and course that strongly distinguishes them from their ovarian counterparts. (It is noteworthy that no adult‐type granulosa cell tumors have been described in the testis to date.) Recent molecular analyses also point to genetic differences between testicular and ovarian juvGCTs, with a high proportion of monosomy 10 in testicular tumors, but not ovarian tumors.^23^ From a clinical perspective, testicular juvGCTs only present in the first year of life, are restricted to the testis, and do not relapse. In fact, we recently described a neonate with a diffuse, bilateral infiltration by a juvGCT, which resolved during endocrine treatment with LH‐RH blockage.^24^ This again indicates the small malignant potential of juvGCTs in the testis, and we even may speculate whether these might constitute developmental tumors with a tendency to spontaneous regress in the first year of life if they are not diagnosed beyond infancy. Therefore, future strategies for testicular juvGCTs should aim for more organ‐sparing surgery.^25^ In this context, however, it should be noted that other testicular tumors in this age group, especially yolk sac tumors, may have an increased risk of relapse after incomplete resection. Therefore, organ‐sparing surgery should not be attempted in infants who have AFP levels above the age‐related reference levels or even in those who have rising AFP levels.^25^ In questionable cases, intraoperative biopsy could help clarify the diagnosis. A comparable approach has also been discussed by the US Testis Tumor Working Group.^25^ Considering the case report of bilateral tumors, and the good overall prognosis, it also may be discussed whether an approach with LH‐RH blockage could be justified.^24^ If juvGCTs of the testis were really hormone‐dependent, tumor shrinkage could be expected within the first weeks of treatment without a risk of progression into the lymph nodes.^24^ Ideally, the testis might even be spared by a partial resection. In summary, testicular juvGCT constitutes an important differential diagnosis of testicular tumors in neonates and young infants, and testis‐sparing strategies should strongly be considered in patients with juvGCTs.

In contrast, organ‐sparing surgical strategies should be undertaken with caution in ovarian juvGCTs. Of note, three of four patients who relapsed initially underwent tumor enucleation or partial ovariectomy, indicating that organ‐sparing surgery may be a potential risk factor in ovarian juvGCTs (Table 5). However, these patients also had another important risk factor: high mitotic activity. Thus, in case of organ‐sparing surgery and a histologic finding of high mitotic activity, second‐look surgery and ovariectomy should be recommended to improve local tumor control. In fact, tumor biology, reflected here by a high mitotic index, appears to emerge as a prognostic factor even in low‐stage tumors (Table 5). Therefore, it should be discussed whether tumors—even at stage IA—should be selected for adjuvant chemotherapy if histologic features, such as high mitotic rate, anaplasia, or lymph vessel invasion, are detected. However, the current results are too preliminary to establish that these biologic risk factors can be overcome with adjuvant chemotherapy. Ideally, the pooling of national registry data into an international data base could address this question, whether adjuvant chemotherapy for patients who have low‐stage tumors with a high mitotic rate might be associated with a lower rate of tumor progression.^26^

In contrast, the implementation of adjuvant chemotherapy for patients who have stage IC2, IC3, or higher stage juvGCTs has been associated with a significant reduction of relapses (Table 4). In the earlier cohort, relapses were reported in more than one half of patients and almost inevitably were fatal (Table 7).^14^, ^15^ Conversely, patients who had only intraoperative tumor spillage could be followed without an increased risk of relapse (Table 4), supporting the chosen therapy stratification.

**TABLE 7:** Comparison between the historical German Maligne Keimzelltumoren cohort and the current cohort: Stage, use of adjuvant chemotherapy, and outcome.

FIGO stage	German MAKEI cohort (Schneider et al.^14^)^a^			STEP 2024		
	No.	Chemotherapy, %	Overall survival, %^a^	No.	Chemotherapy, %	Overall survival, %
IA	24	0	100	52	4	98
IC1	7	43	100	14	7	100
IC2/IC3	8	38	50	17	76	100
II/III	6	100	83	5	100	80
IV	0	—	—	0	—	—
Sum	45	27	88	89	24	97

Abbreviations: FIGO, International Federation of Gynecology and Obstetrics; MAKEI, Maligne Keimzelltumoren; STEP, German Registry for Rare Pediatric Tumors.

^a^ Updated in 2020: four of six patients who relapsed died 10–20 months after the diagnosis of relapse, and one patient developed a fatal relapse at 123 months of follow‐up, resulting in a 5‐year survival rate of 50% for the group with stage IC2 disease and 88% for the complete cohort.

In the initial historic MAKEI cohort, 27% of patients were treated with chemotherapy, resulting in six relapsed patients among 45 patients and five deaths.^14^ In the current series, we have observed four relapses and two deaths among 89 patients, whereas the rate of patients receiving chemotherapy is comparably low. This strongly indicates that the risk stratification and selection of patients for chemotherapy have worked better in the recent study, meaning that low‐risk patients were safely spared chemotherapy while patients at risk were successfully selected for chemotherapy, with a low relapse rate. In total, the overall survival rate of this cohort fits well or even exceeds that reported by other national study groups with cohorts of significant size.^20^

This strict distinction between stage IC1 and IC2/3 tumors, i.e., preoperative versus intraoperative spread, has not been used for therapy stratification in all national study groups. In fact, most national groups would still consider chemotherapy for stage IC1 tumors. Therefore, we appreciate that more study groups have now started adopting the strategy to selectively restrict chemotherapy to patients with stage IC2 or later stage tumors.^21^, ^27^

It should be emphasized that this strategy is only justifiable if reference structures have been established. This means that surgical reports are critically reviewed centrally according to uniform criteria and a reference pathologic assessment is performed. This is the only way to ensure that the prognostic factors recognized as relevant in this study are safely identified and that the treating clinics can be advised accordingly.

In summary, the current study demonstrates that, even for extremely rare tumors like juvGCTs with less than 10 cases per year in Germany, therapeutic strategies can be developed and further optimized based on registries and consultation structures. In the future, biologic parameters will probably also be used for therapy stratification. International networking will make it possible to further optimize treatment concepts for juvGCTs with the primary aim of reducing treatment for low‐risk patients.

## AUTHOR CONTRIBUTIONS

**Dominik T. Schneider:** Conceptualization, data curation, formal analysis, writing–original draft, methodology, investigation, supervision, project administration, writing–review and editing, software, funding acquisition, resources, and validation. **Andrea Witowski:** Formal analysis, writing–original draft, writing–review and editing, data curation, software, and investigation. **Michael Abele:** Data curation, formal analysis, writing–review and editing, investigation, validation, funding acquisition, and project administration. **Martin Benesch:** Data curation, formal analysis, and writing–review and editing. **Benedikt Bernbeck:** Data curation, formal analysis, writing–review and editing, resources. supervision. and investigation. **Tabea Blessing:** Formal analysis, resources, funding acquisition, validation, writing–review and editing, and project administration. **Bastian Brummel:** Formal analysis, writing–review and editing, and data curation. **Gabriele Calaminus:** Conceptualization, writing–review and editing, and resources. **Ulrich Gobel:** Conceptualization, data curation, writing–review and editing, investigation, resources, funding acquisition, validation, and supervision. **Norbert Graf:** Formal analysis, writing–review and editing, supervision, methodology, and resources. **Christian Vokuhl:** Formal analysis, data curation, investigation, supervision, writing–review and editing, and validation. **Kris Ann P. Schultz:** Conceptualization, writing–review and editing, and supervision. **Ines B. Brecht:** Conceptualization, writing–review and editing, project administration, supervision, investigation, methodology, data curation, formal analysis, resources, funding acquisition, and validation.

## CONFLICT OF INTEREST STATEMENT

The authors disclosed no conflicts of interest.

## Data Availability

The data that support the findings of this study are available on request from the corresponding author. The data are not publicly available due to privacy or ethical restrictions.
